# Phenotypic and Genotypic Profile of *Enterobacteriaceae* Isolated at a Teaching Hospital in Ghana

**DOI:** 10.1002/mbo3.70127

**Published:** 2025-11-09

**Authors:** Bismark Donkor, Richael Odarkor Mills, Philimon Mwintige, Alberta Bedford Moses, Abigail Asmah Brown, Faustina Halm‐Lai, Oheneba Charles Kofi Hagan

**Affiliations:** ^1^ Department of Microbiology and Immunology, School of Medical Sciences, College of Health and Allied Sciences University of Cape Coast Cape Coast Central Region Ghana; ^2^ Department of Biomedical Sciences, School of Allied Health Sciences, College of Health and Allied Sciences University of Cape Coast Cape Coast Central Region Ghana; ^3^ Department of Microbiology Cape Coast Teaching Hospital Cape Coast Central Region Ghana; ^4^ Laboratory Department Cape Coast Teaching Hospital Cape Coast Central Region Ghana; ^5^ Department of Medical Biochemistry, School of Medical Sciences, College of Health and Allied Sciences University of Cape Coast Cape Coast Central Region Ghana

**Keywords:** AMR, *Enterobacteriaceae*, plasmids, virulence factors, WGS

## Abstract

Antibiotic resistance in *Enterobacteriaceae* continues to rise, and its implications for healthcare delivery have intensified. We investigated the genetic basis of antimicrobial resistance (AMR), virulence genes, and associated plasmids in *Enterobacteriaceae* isolates from a teaching hospital in Ghana. Antimicrobial susceptibility testing was performed using archived isolates. Whole genome sequencing was performed on a subset of the isolates, that were either multidrug resistant or extended‐spectrum β‐lactamase (ESBL)‐producing. Bioinformatics analyses were performed for speciation, identification of AMR and virulence genes, and associated plasmids. The 100 *Enterobacteriaceae* isolates included in this study showed high phenotypic resistance to ß‐lactams and susceptibility to aminoglycosides. Nineteen of the 20 WGS isolates were genotypically identified using the housekeeping genes as *Escherichia coli* (8/20, 40%), *Klebsiella pneumoniae* (8/20, 40%), *Enterobacter cloacae* (2/20, 10%), and *Salmonella enterica* (1/20, 5%). These strains harboured 139 unique antibiotic resistance genes (ARGs) encoding resistance against ß‐lactams (64/139), aminoglycosides (23/139), fluoroquinolones (45/139), tetracyclines (37/139), phenicols (27/139), and sulphonamides (9/139). Subsequent AST revealed that (74/79, 94%) were ESBL producers, and (9/79,11%) were carbapenem resistant *Enterobacteriaceae* (CRE). The isolates expressed 8 categories of virulence factors (VFs), including effector delivery systems, adherence, and metabolic factors. Additionally, 26 unique plasmid replicons of both the I‐complex and colicin plasmids were detected. We observed phenotypic and genotypic evidence of antimicrobial resistance to common antibiotics in the isolates driven by *CTX‐M‐15* and in some *K. pneumoniae*, the *NDM‐1*. These findings highlight the urgent need to improve antibiotic stewardship, surveillance and control at the hospital.

## Graphical abstract

This study identified a significant prevalence of antimicrobial resistance determinants within *Enterobacteriaceae* isolates, with particular emphasis on those producing extended‐spectrum β‐lactamases (ESBL). These isolates harbored diverse antibiotic resistance genes (ARGs), plasmid replicons, and virulence factors (VFs), highlighting the imperative for enhanced genomic surveillance and antimicrobial stewardship in hospital settings.

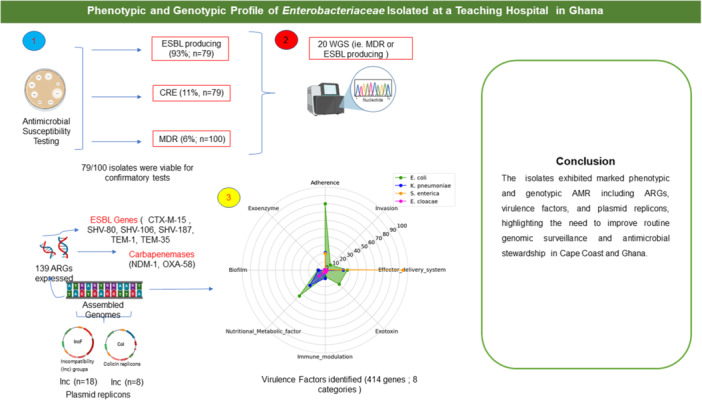

## 1 Introduction

The family *Enterobacteriaceae* is widely distributed in nature, particularly in the environment and gastrointestinal tracts of animals. They are Gram‐negative, non‐spore‐forming, facultative anaerobes, reduce nitrate to nitrite, and ferment glucose (Octavia and Lan 2014; Janda and Abbott 2021; Jenkins et al. 2017). Although the majority of these are commensals, some genera have an impact on human and animal health. These include *Escherichia coli, Klebsiella spp, Citrobacter spp, Salmonella spp*, and *Enterobacter spp (*Janda and Abbott 2021; Tacconelli et al. 2018; Renata Silva‐Lacerda et al. 2015).

The emergence and persistence of antimicrobial resistance (AMR) in the *Enterobateriaceae* to antimicrobials of various classes, especially extended‐spectrum ß‐lactams, carbapenems, and fluoroquinolones, in clinical settings have hampered global healthcare delivery, especially in lower‐ and middle‐income countries (LMICs) 2020 (Walsh et al. 2023; Lynch et al. 2021). A systematic review and meta‐analysis by Bezabih et al. found a 21.1% in the global antibiotic resistance *Enterobacteriaceae* (ARE) rate, particularly in isolates that are ESBL carriage from 2003 to 2018 in the healthcare settings (Bezabih et al. 2022a). Other studies have highlighted the prevalence of the CTM‐X‐15 gene, a type of ESBL gene that is dominant among pathogenic *Enterobacteriaceae* (Irenge et al. 2019; Asare Yeboah et al. 2024). With regard to carbapenem resistance, the pooled prevalence globally is higher than the data available in Africa. They reported NDM, IMP, and VIM types as the main drivers of carbapenem‐resistant *Enterobacteriaceae* (CRE), especially NDM‐1. However, data present in Ghana reveal low harborage of NDM‐1 (Bachelle et al. 2024; Ayibieke et al. 2018). Nonetheless, CRE in Ghana continues to rise. Mills et al. and Ayibieke et al. reported that carbapenem‐hydrolyzing class D β‐lactamases, mainly OXA‐type genes, were observed among *Enterobacteriaceae* in Ghana (Ayibieke et al. 2018; Mills et al. 2024).

Aside from antibiotic‐resistant genes (ARGs), these bacteria harbor genes of virulence factors (VFs). VFs are important for *Enterobacteriaceae* to successfully colonize and survive in their hosts (Bujňáková et al. 2022; Dekker et al. 2021), eventually causing infections (Lynch et al. 2021; Irfan et al. 2022; Tilahun et al. 2021). VFs encompass diverse mechanisms, such as host cell adhesion (e.g., pili and adhesins), tissue invasion (e.g., invasins and hyaluronidase), toxin production (e.g., exotoxins and endotoxins), immune evasion (e.g., capsules and antigenic variation), enzymes facilitating spread (e.g., coagulase and proteases), iron acquisition (e.g., siderophores), secretion systems (e.g., Type III/VI), intracellular survival strategies, and antiphagocytic (Bujňáková et al. 2022; Leitão 2020; Liu et al. 2022a). Surveillance studies on VFs among *Enterobacteriaceae* revealed an abundance of VFs in ARE (Bujňáková et al. 2022; Leitão 2020; Payne and Neilands 1988; Pakbin et al. 2021).

Both ARGs and VFs are harbored within bacterial chromosomes and mobile elements such as plasmids, transposons, and integrons (Lynch et al. 2021; Aleshina et al. 2024; Partridge 2015; Toombs‐Ruane et al. 2017). Present data reveal that they are usually borne on plasmids (Hawkey et al. 2022; Carattoli et al. 2021), the dominant mobile genetic elements in antibiotic‐resistant bacteria (Harris et al. 2023; Wang and Dagan 2024). Incompatibility complex (I‐complex) plasmids, mostly consisting of resistance and fertility groups, are prevalent in ARE (Hawkey et al. 2022; Rozwandowicz et al. 2018; Atala 2020). IncF, IncC, and IncX have been reported to be the most I‐complex plasmids involved in the transmission of AMR among *Enterobacteriaceae (*Carattoli et al. 2021; Ma et al. 2023; Yu et al. 2022 Nov 16). In addition to I‐complex plasmids, *Enterobacteriaceae* also harbor colicin plasmids. These plasmids produce bacteriocins, which have been reported to inhibit other bacteria and are implicated in ARGs and VFs transmission (Ghanim et al. 2023). They are usually present in *Enterobacteriaceae*, especially *E. coli* and *Klebsiella spp* (Atala 2020; Ghanim et al. 2023; Marković et al. 2022a).

Previous studies have uncovered a high record of *Enterobacteriaceae* infections in the Cape Coast Teaching Hospital (CCTH) (Dakorah et al. 2022; Anning et al. 2022); however, data on the genetic basis of ARE, especially those that are ESBL‐producing, are revealed at the hospital due to the poor antimicrobial resistance surveillance in Ghana. This study combined phenotypic and genomic approaches to comprehensively characterize the AMR profiles of *Enterobacteriaceae* isolated from patients at CCTH, providing robust genetic evidence to support existing surveillance data. Through phenotypic identification and antimicrobial susceptibility testing (AST), we determined the prevalence of multidrug‐resistant (MDR) strains, extended‐spectrum β‐lactamase (ESBL) producers, and CRE. Additionally, whole genome sequencing (WGS) of 20 ESBL‐producing isolates was performed to characterize their taxonomy, antimicrobial resistance genes (ARGs), VFs, and harbored plasmid replicons.

## 2 Materials and Methods

### 2.1 Study Design and Site

This was a retrospective study of archived *Enterobacteriaceae* isolates from patients attending CCTH, a tertiary‐level health facility located in the Cape Coast Metropolitan Area of the Central Region of Ghana. The hospital is a 400‐bed capacity facility that serves as a referral center for the central, western, western east, and eastern regions of Ghana. The microbiology laboratory at the hospital routinely conducts culture and sensitivity testing for microorganisms isolated from various specimen sources, including blood, urine, abscess, stool, wound swabs, and high vaginal and endocervical swabs. Specimens received from the various departments within the hospital, including *Enterobacteriaceae* isolates archived from 2020 to 2023, were included in this study. The departments included were outpatient department (OPD), pediatric ward, emergency wards, intensive care unit (ICU), and obstetrics and gynecology (O/G).

### 2.2 Bacterial Isolates

Bacterial isolates of the *Enterobacteriaceae* family were retrieved from storage and phenotypically identified using standard biochemical tests, including citrate, oxidase, indole, urease, and triple sugar iron (TSI).

### 2.3 Antimicrobial Susceptibility Testing (AST)

AST was performed for the isolates using the following antimicrobial agents: ß‐lactams: ampicillin [AMP 10 mcg], ceftriaxone [CTR, 30 mcg], cefotaxime [CTX 30 mcg], cefuroxime [CXM 30 mcg], and meropenem [MRP, 10 mcg]; fluoroquinolones: ciprofloxacin [CIP 5 mcg], levofloxacin [LEV 5 mcg], and ofloxacin [OF 5 mcg]; aminoglycosides: amikacin [AMK 30 mcg] and gentamicin [GEN 10 mcg]; sulfonamide: cotrimoxazole [COT 25 mcg]; tetracycline [TET 30 mcg]; and chloramphenicol [CHL 30 mcg]. AST was performed using the Kirby–Bauer disk diffusion method, and the breakpoints were interpreted according to the Clinical and Laboratory Standard Institute (CLSI) 2020 guidelines (CLSI 2020). Briefly, pure colonies of the cultured isolates were picked from agar culture plates and inoculated into peptone broth to achieve turbidity equivalent to 0.5 McFarland standards. Using a sterile cotton bud, a swab of the bacterial suspension was streaked onto the entire surface of a freshly prepared Mueller–Hinton agar (MHA) plate. Antibiotic discs were placed on the MHA in the plates within 15 min of bacterial inoculation. The plates were then incubated at 37°C for 24 h. Subsequently, the zone of inhibition was measured in millimeters using a meter rule and interpreted as sensitive, intermediate, or resistant according to the CLSI 2020 guideline (CLSI 2020). Based on the AST findings, we classified the isolates resistant to 3 or more classes of antibiotics as MDR (Magiorakos et al. 2012) and ESBL‐producing according to the CSLI 2020 guideline.

### 2.4 ESBL Producers Screening

Isolates exhibiting an inhibitory zone diameter ≤ 27 mm upon exposure to cefotaxime (30 mcg) and ≤ 25 mm when tested with ceftriaxone (CTR: 30 mcg) according to the (CLSI 2020) guide were identified as possible ESBL producers. ESBL confirmatory tests were performed (*n* = 79) using ceftazidime (CAZ:10 mcg) and ceftazidime‐clavulanic acid (CAL: 40 mcg). Isolates that exhibited an inhibitory zone diameter of ≤ 17 mm upon exposure to ceftazidime (30 mcg) and difference around CAL to CAZ ≥ 5 mm when compared were confirmed as ESBL producers.

### 2.5 Whole Genome Sequencing (WGS)

Twenty ESBL‐producing isolates were selected for WGS. Genomic DNA (gDNA) was extracted from the bacteria using the Quick‐DNA Mini Prep Plus Kit™ (Zymo Research, Irvine®, United States) according to the manufacturer's instructions. WGS was performed at the WACCBIP NGS Laboratory (Accra, Ghana) using an Illumina MiSeq® (Illumina, Inc.®, San Diego, USA). DNA quality and quantity were determined using a Qubit™ 4.0 fluorometer (Thermo Fisher Scientific®, Waltham, USA). Short read sequencing was performed using (2 × 250 bp) paired end (PE) sequencing with the MiSeq® Reagent Kit v3 (600 cycles). For each sample, 100 ng total DNA was used for library preparation. Sequencing libraries were prepared from the enriched DNA using the Illumina® DNA Prep (Illumina) library preparation kit from the enriched DNA, following the manufacturer's instructions. Using the Nextera XT Index Kit v2™ (Illumina), distinct indices and Illumina sequencing adapters were attached to each library according to the manufacturer's instructions. Subsequently, each library was purified using Agencourt AMPure XP beads (Beckman Coulter®). Agilent 4200 TapeStation™ (Agilent®) was then used to check the expected size distribution and quality of the library. The library concentrations were measured using a Qubit™ 4.0 fluorometer (Life Technologies™). Based on the TapeStation and Qubit results, the barcoded libraries were normalized and pooled at an equimolar concentration. The combined library was diluted to 18 pM, spiked with 5% Phix™ (v3), and then sequenced at a depth of 50X and a coverage of 30X.

### 2.6 Bioinformatics Analyses

Adapters and low‐quality reads of (*n* = 20) PE reads were trimmed using Trimmomatic (v0.39) (https://github.com/usadellab/Trimmomatic), setting Phred score at 33, ILLUMINACLIP: Nextera‐PE. fa: 2:30:10, LEADING:3, TRAILING:3 and MINLEN:22. The PE trimmed reads (*n* = 20) were assembled using SPAdes Genome Assembler (v4.0.0) (https://github.com/ablab/spades), and quality assessment was performed using QUAST (v5.2.0) (https://github.com/ablab/quast). Multilocus sequence typing (MLST) (https://github.com/tseemann/mlst), which scans contigs against traditional PubMLST typing schemes in addition to ribosomal MLST (https://pubmlst.org/species-id), was performed to validate the identity of the isolates. Concurrently, each draft genome was screened for the presence of AMR genes using Abricate (https://github.com/tseemann/abricate), which hosts (CARD (v3.2.6), PlasmidFinder, and VFDBS) (accessed June 2024). The resistance and virulence genes were determined using CARD (Alcock et al. 2020; Jia et al. 2017) and VFDBS (Liu et al. 2022a), respectively. Assembled contigs were further assessed to identify plasmid genes using PlasmidFinder (Carattoli et al. 2014) to understand the transmission of AMR genes within the *Enterobacteriaceae* population at the hospital. Genes were selected based on coverage and identity equal to or greater than a threshold of 95%.

### 2.7 Data Analysis

Data visualization was conducted using Python (v3.11) in Jupyter (v3.6.3) via the Anaconda Navigator (v2.4.6), employing Matplotlib, pandas, plotly express, and seaborn libraries. Heatmaps illustrating antibiotic susceptibility patterns, sunburst presenting ARGs found in isolates and against antibiotics, and radar plot depicting virulence factor distributions among *Enterobacteriaceae* isolates were generated using the same libraries. Statistical analyses were performed using Jamovi (v2.3.28). Descriptive statistics summarized patient demographics and clinical characteristics, with median and interquartile ranges. Cohen's Kappa was used to evaluate interrater reliability between phenotypic, MLST, and rMLST identification methods, implemented via the ClinicoPath module in Jamovi.

## 3 Results

### 3.1 Socio‐Demographics and Clinical Data

The majority of bacterial isolates (70%) were from females, with 34% and 36% being within the age categories of 20–29 and 40–69, respectively. The ages ranged from 8 days to 85 years, with a median age of 40 years and an interquartile range of 35 years. Of the 100 samples, 51% and 10% were specimens referred from the OPD and accident and emergency department (A&E), respectively. Other departments, including the intensive care unit (ICU), female surgical ward (FSW), male surgical ward (MSW), and obstetrics and gynecology (O/G), contributed to 13% of the isolates. The majority of the isolates (44%) were obtained from urine samples as presented in Table 1.

**Table 1:** Demographics and clinical characteristics of isolates corresponding to patients at CCTH.

Category	Subcategory	Percentage (%) *n* = 100
Age in years	≤ 12 years	12.0
	13–19	2.0
	20–39	34.0
	40–69	37.0
	≥ 70	15.0
Sex	Female	70.0
	Male	30.0
Department	OPD	51.0
	Other Departments	13.0
	A/E	10.0
	FMW	8.0
	MMW	7.0
	ETAT	6.0
	PMW	5.0
Specimen source	Urine	44.0
	Sputum	14.0
	HSV	12.0
	Wound swab	12.0
	Other sources	10.0
	Blood	8.0

Abbreviations: A/E, accident and emergency; ETAT, emergency triage, assessment and treatment; FMW, female medical ward; MMW, male medical ward; OPD, outpatient department; PMW, pediatric medical ward.

### 3.2 Antimicrobial Susceptibility Patterns

**Figure 1:**
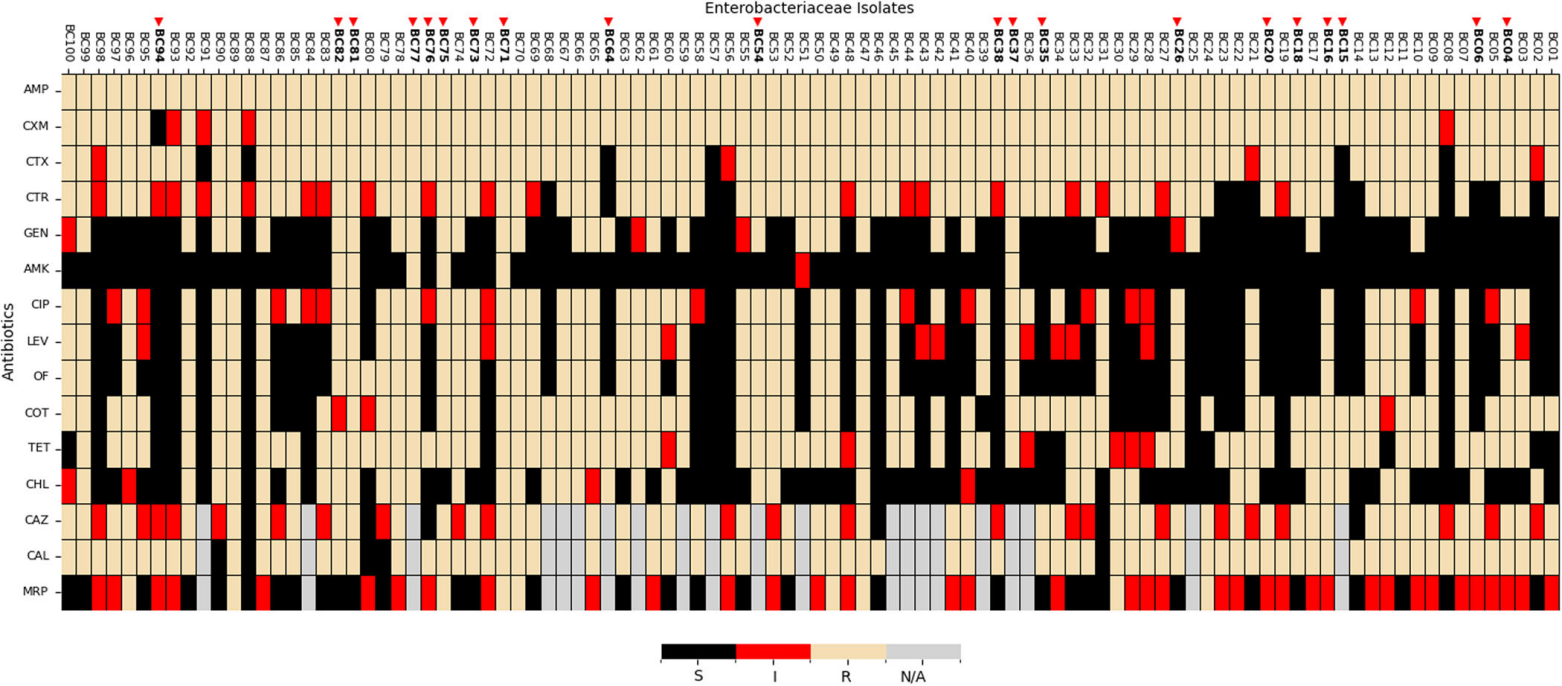
Antibiotic susceptibility profiles (phenotype) of *Enterobacteriaceae* bacteria Isolates. The heatmap displays antibiotic susceptibility patterns across various *Enterobacteriaceae* isolates for different antibiotics. Each row represents a specific antibiotic, while each column corresponds to an isolate. The color‐coding indicates the susceptibility categories: black: susceptible (S), red: intermediate resistance (I), beige: resistant (R), gray: data not available (N/A). The antibiotics tested were (ß‐lactams: Ampicillin [Amp], Ceftriaxone [CTR], Cefotaxime [CTX], Ceftazidime [CAZ], Meropenem [MRP], and Cefuroxime [CXM]; Fluoroquinolones: Ciprofloxacin [CIP], Levofloxacin [LEV], and Ofloxacin [OF]; Aminoglycosides: Amikacin [AMK] and Gentamicin [GEN]; Sulfonamides: Cotrimoxazole [COT]; Tetracycline [TET]; Chloramphenicol [CHL]; and Ceftazidime‐Clavulanic acid [CAL]).

Of the hundred (100) isolates, 6% were MDR. ESBL testing was successful for 79 isolates, out of which 74(93.7%) were ESBL‐producing. Approximately 93%, 69%, and 63% of the 100 isolates were susceptible to amikacin, chloramphenicol, and gentamicin, respectively. However, all isolates were resistant to ampicillin (Figure 1). Meropenem sensitivity testing was performed for the 79 isolates, of which 40.51% (32/79), 48.10% (38/79), and 11.39% (9/79) were susceptible, intermediate, or resistant, respectively. This suggests that the isolates are mostly susceptible to meropenem in the hospital.

### 3.3 Whole Genome Assembly

Computation of the total read count and quality metrics of the assemblies (Table A5) was consistent and of high quality, with mean number of contigs, N50, and assembly size as 647, 212,022, and 7,025,968, respectively. Tseeman/mlst analysis predicted *E. coli* (8/20, 40%), *K. pneumoniae* (8/20, 40%), *E. cloacae* (2/20, 10%), and *S. enterica* (1/20, 5%). Additionally, (1/20, 5%) isolate initially identified as *E. coli* by biochemical tests was identified as *Pseudomonas aeruginosa*, which belongs to the family *Pseudomonadaceae* (Table A1). Agreement between the phenotypic and genotypic identification of the *Enterobacteriaceae* isolates was moderate (0.60), with a kappa value of 0.47. The statistical difference between the phenotypic and genotypic identification of the *Enterobacteriaceae* isolates was significant (*p*‐value < 0.001). This suggests that the phenotypic and genotypic methods, including MLST and rMLST, do not consistently agree with their classification of bacterial isolates.

### 3.4 Resistome

CARD revealed 139 unique ARGs that would confer resistance to the following classes of antibiotics: ß‐lactams (64/139), fluoroquinolones (45/139), aminoglycosides (23/139), phenicol (27/139), tetracycline (37/139), and sulfonamide (9/139) (Figure 3; Table A3). High diversity of ARGs was observed in *E. coli* (130/139), followed by *K. pneumoniae* (111/139), *E. cloacae* (9/139), and *S. enterica* (8/139) (Figure 4, Table A3). Isolate IDs BC82 (*E. coli*) harbored the most ARGs (74/139), whereas the lowest was detected in BC94 (*K. pneumoniae)* (6/139). *CTX‐M‐15*, an ESBL gene, was the most commonly observed ARG, detected in (13/19) isolates (Table A2). Nine ARGs, including *APH (3')‐Ia, Erm (*Ares‐Arroyo et al. 2021 *), EC‐15, Escherichia_coli_emrE, golS, mdsC, MdtK, QnrD1*, and *SHV‐80*, were recorded as the least common occurring ARGs among the isolates. Each was observed once in a single isolate. Several variants of MDR genes (91/139) were observed in the isolates, especially in *E. coli* (65/91), followed by *K. pneumoniae* (44/91), *S. enterica* (5/91), and *E. cloacae* (1/91). They were observed to have conferred resistance, especially to 4 classes of antibiotics, including ß‐lactams, fluoroquinolones, tetracycline, and chloramphenicol.

### 3.5 Virulome

**Figure 2:**
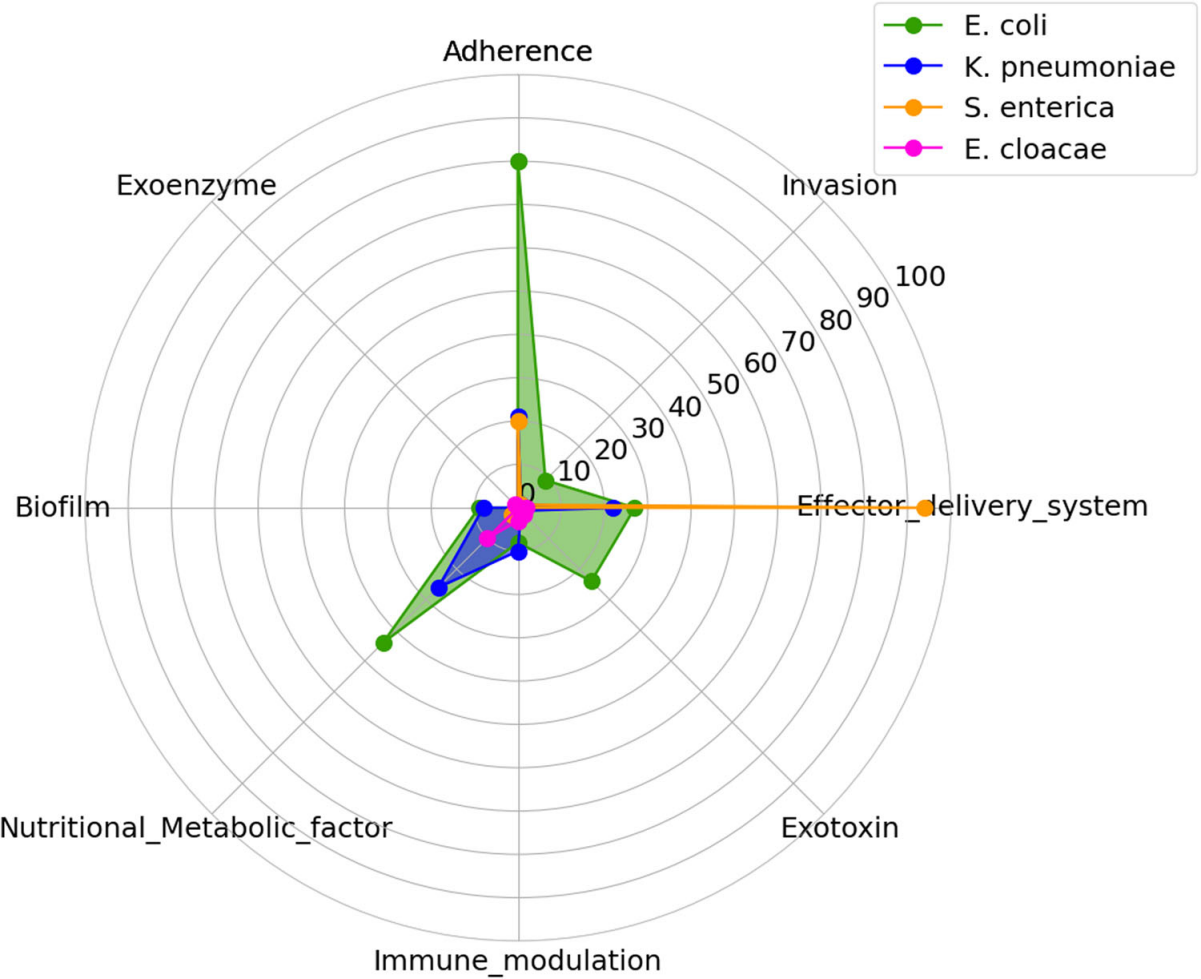
Virulence factor distribution among *Enterobacteriaceae* bacterial species. This radar chart illustrates the distribution of eight virulence factors (Adherence, Invasion, Effector delivery system, Exotoxin, Immune modulation, Nutritional/Metabolic factor, Biofilm, and Exoenzyme) across four *Enterobacteriaceae* bacterial species: *E. coli* (green), *K. pneumoniae* (blue), *S. enterica* (orange), and *E. cloacae* (pink). Each axis represents a virulence factor, with the distance from the center showing its strength or pathogenic and AMR contribution in each species. *E. coli* stands out in Adherence and Nutritional/Metabolic factors, while *S. enterica* is strong in the Effector delivery system. The other species show varying levels of these factors, reflecting their different pathogenic profiles.

Using VFDBS, several VFs previously described in the *Enterobacteriaceae* family (*E. coli, K. pneumoniae, E. cloacae*, and *S. enterica*) were detected. In total, 414 unique virulence genes were detected (Table A4) and categorized into 8 major groups and 17 subcategories, as detailed in Table 2 and Figure 2. The predominant VF categories included adhesins, invasins, effector delivery systems, exotoxins, immune evasins, nutrient and metabolic factors, biofilms, and exoenzymes. Most of these VFs were observed in *E. coli* isolates, followed by *K. pneumoniae, E. cloacae*, and *S. enterica*. Adhesin‐associated VFs, which are essential for bacterial attachment to host cell mucosae (Abraham et al. 1988), were predominantly identified in *E. coli* and *K. pneumoniae*. Invasion‐related VFs, which facilitate host cell penetration, were detected in all four genera. Effector delivery systems, including T2SS and T3SS, are known for injecting bacterial proteins into host cells to manipulate immune signaling, cell death, and nutrient acquisition pathways (Nans et al. 2015; Pinaud et al. 2018) and were also observed in all the genera, principally in the *S. enterica* isolate, as shown in Figure 2. Exotoxins, particularly membrane‐acting, intracellular active toxins, were observed in *E. coli*, *K. pneumoniae*, and *E. cloacae* isolates; however, none were observed in the *S. enterica isolate*. Similarly, immune evasion VFs, including antiphagocytosis and complement evasion mechanisms, were present in all *Enterobacteriaceae* isolates except *E. cloacae*. Nutritional and metabolic VFs were mostly present in *E. coli*, followed by *K. pneumoniae* and *E. cloacae*. These factors, particularly those involved in metal uptake, metabolic adaptation, and iron sequestration, are crucial for bacterial survival and proliferation in host environments (Payne and Neilands 1988; Shealy et al. 2021). Biofilm‐associated VFs, which contribute to antibiotic resistance, are confined to *E. coli* and *K. pneumoniae*. Meanwhile, proteases that degrade exogenous proteins, enhancing bacterial growth and metabolism (Liu et al. 2022b), were uniquely observed in *E. cloacae*. Bacterial survival in host environments plays an important role in persistent infections (Grant and Hung 2013). *Enterobacteriaceae* strains harboring these VFs can subvert the host immune defenses, leading to recurrent infections (Bujňáková et al. 2022; Folgori et al. 2021). The diversity of VFs identified in the *Enterobacteriaceae* isolates accentuates their adaptability within host environments, enhancing their ability to evade antimicrobial defenses and establish infections.

**Table 2:** Virulence factors and sub‐categories present among *Enterobacteriaceae* bacterial isolates.

Virulence factor genes			
Organism	Category	Subcategory	No. unique VFs
*E. coli*	Adherence		
		Fimbrial adhesin	77
		Non‐fimbrial adhesin	2
	Invasion		9
	Effector delivery system		
		Type II secretion system (T2SS)	11
		Type V secretion system (T5SS)	2
		Type VI secretion system (T6SS)	14
	Exotoxin		
		Membrane‐acting toxin	5
		Intracellularly active toxin	19
	Immune modulation		
		Antiphagocytosis	1
		Complement evasion/Serum resistance	1
		Inflammatory signaling pathway	6
	Nutritional/Metabolic factor		
		Metal uptake	44
	Biofilm		
		Biofilm formation	7
		Quorum sensing	2
*K. pneumoniae*	Adherence		
		Fimbrial adhesin	21
	Invasion		1
	Effector delivery system		
		Type II secretion system (T2SS)	1
		Type VII secretion system (T7SS)	21
	Exotoxin		
		Intracellularly active toxin	1
	Immune modulation		
		Complement evasion/Serum resistance	5
		Inflammatory signaling pathway	5
	Nutritional/Metabolic factor		
		Metal uptake	25
		Metabolic adaptation	1
	Biofilm		
		Biofilm formation	5
		Quorum sensing	3
*S. enterica*	Adherence		
		Fimbrial adhesin	19
		Non‐fimbrial adhesin	1
	Invasion		1
	Effector delivery system		
		Type III secretion system (T3SS)	85
		Type VI secretion system (T6SS)	9
	Nutritional/Metabolic factor		
		Metal uptake	2
*E. cloacae*	Effector delivery system		
		Type VII secretion system (T7SS)	2
	Exotoxin		
		Membrane‐acting toxin	2
	Immune modulation		
		Antiphagocytosis	3
	Nutritional/Metabolic factor		
		Metal uptake	10
	Exoenzyme		
		Protease	1

**Figure 3:**
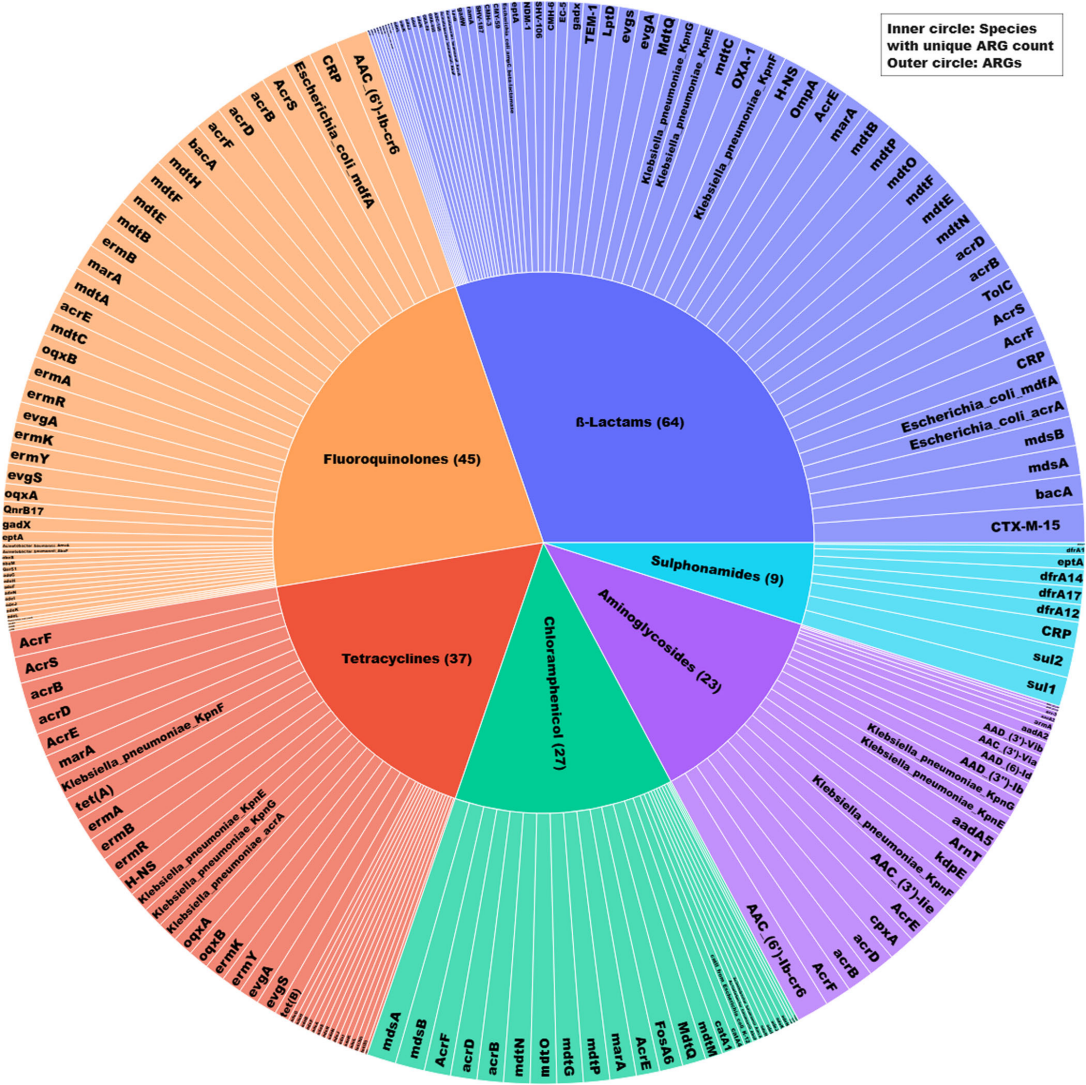
Distribution of ARGs conferring resistance to major antibiotic classes. The inner ring represents each class, and the outer ring represents individual ARGs. ARGs conferring resistance against β‐lactams were the most represented (Sawa et al. 2020), followed by fluoroquinolones (Shealy et al. 2021) and tetracyclines (CLSI 2020) (see Table A3).

**Figure 4:**
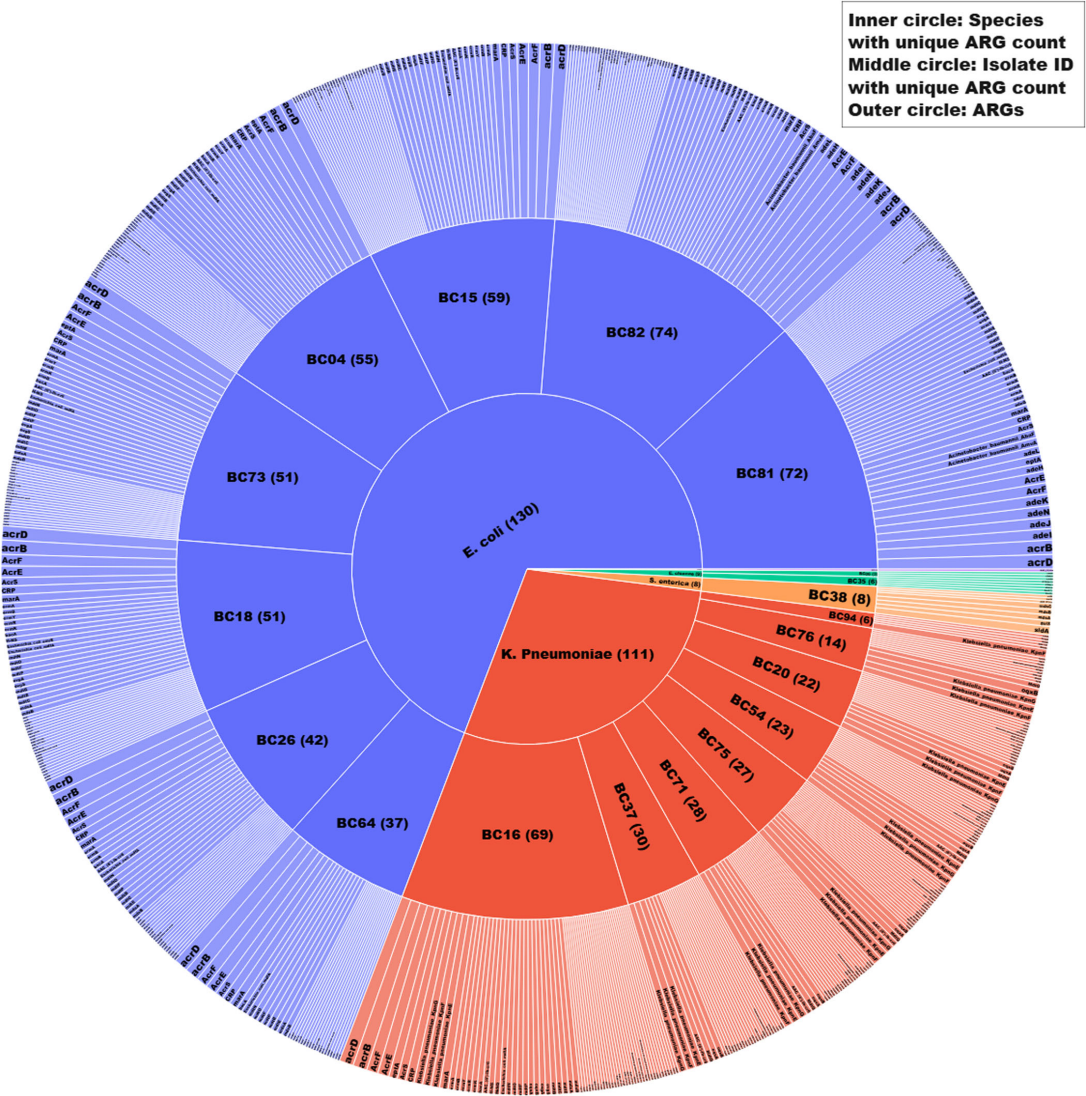
Distribution of ARGs among bacterial isolates, with the inner circle representing species, the middle circle showing individual isolates, and the outer circle depicting detected ARGs. The visualization highlights *E. coli* and *K. pneumoniae* as the predominant species, each harboring diverse ARGs (see Table A3).

### 3.6 Plasmidome

The results from Plasmid Finder revealed a diverse array of plasmid types classified under Incompatibility (Inc) complex plasmids and colicin (Col) plasmids (Table 3). The number of plasmid replicons per isolate significantly varied. *K. pneumoniae* exhibited the highest diversity of plasmids (18/26), followed by *E. coli* (9/26) and *E. cloacae* (5/26). The highest plasmid diversity was observed in isolate BC37 from *pneumoniae* (8/26), whereas BC82 (*E. coli*) and BC26 (*K. pneumoniae*) exhibited the lowest (1/26 each). Only *S. enterica* in this collection harbored no plasmid replicons. Among the Inc plasmids that are mostly observed in *Enterobacteriaceae* as vehicles for AMR transmission (Rozwandowicz et al. 2018), the IncF subgroups were the most frequently detected. *IncFIB (AP001918) _1* was the most prevalent, identified in eight isolates, followed by *IncFIB(K)_1* and *IncFIA_1*, which were each found in 7 isolates. *IncFII_1, IncHI1B(pNDM‐MAR) _1*, and *IncR_1* were detected in 3 isolates each. Plasmids such as *IncC_1, IncFII (*Rozwandowicz et al. 2018 *) _1*, *IncFII(pCoo)_1, IncX4_2*, and *pKPC‐CAV1321_1* were found in only a single isolate each. Colicin plasmids encoding colicins (bacteriocins), proteins that kill other bacteria (Atala 2020), were also distributed across the isolates. They have been reported to contribute to the spread of antibiotic resistance and other VFs among uropathogenic *E. coli* strains and *Klebsiella spp* by co‐integration with other plasmids such as IncC, *IncF, and IncN* (Ghanim et al. 2023; Ares‐Arroyo et al. 2021). *Col156_1* was the most frequently detected gene (Tacconelli et al. 2018). *ColRNAI_1* was present in 3 isolates, whereas *Col (BS512) _1 and Col440I_1* were each observed in 2 isolates. The least common Colicin plasmids, found in single isolates, included *Col (KPHS6) _1, Col3M_1*, and *ColpVC_1*.

**Table 3:** Plasmid replicons present among *Enterobacteriaceae* isolates.

PGs		*Escherichia coli*								*Klebsiella pneumoniae*								*Enterobacter cloacae*		Number of isolates
		BC04	BC15	BC18	BC26	BC64	BC73	BC81	BC82	BC16	BC20	BC37	BC54	BC71	BC75	BC76	BC94	BC06	BC35	
Incompatibility (I) complex Plasmid	*IncC_1*																			**1**
	*IncFIA (HI1)_1*																			**2**
	*IncFIA_1*																			**7**
	*IncFIB (AP001918) _1*																			**8**
	*IncFIB(K)_1*																			**7**
	*IncFIB(K)(pCAV1099‐114)_1*																			**1**
	*IncFIB(pKPHS1)_1*																			**2**
	*IncFIB(pNDM‐Mar)_1*																			**3**
	*IncFII (29)_1*																			**1**
	*IncFII(pCoo)_1*																			**1**
	*IncFII(pKP91)_1*																			**2**
	*IncFII(pRSB107)_1*																			**3**
	*IncFII_1*																			**3**
	*IncHI1B(pNDM‐MAR)_1*																			**3**
	*IncI (Gamma)_1*																			**2**
	*IncR_1*																			**3**
	*IncX4_2*																			**1**
	*pKPC‐CAV1321_1*																			**1**
Colicin plasmid	*Col (BS512)_1*																			**2**
	*Col (KPHS6)_1*																			**1**
	*Col156_1*																			**4**
	*Col3M_1*																			**1**
	*Col440I_1*																			**2**
	*Col440II_1*																			**2**
	*ColRNAI_1*																			**3**
	*ColpVC_1*																			**1**
Total PGs in isolate		**3**	**2**	**4**	**3**	**4**	**3**	**4**	**1**	**7**	**1**	**8**	**5**	**5**	**7**	**3**	**3**	**1**	**4**	*

*Note:* Plasmid replicons detected in *E. coli*, *K. pneumoniae*, and *E. cloacae* isolates are listed by incompatibility (Inc) and colicin plasmid types. Columns BC04–BC94 correspond to individual bacterial isolates, while each row denotes a specific plasmid replicon. Presence of a replicon within an isolate is indicated by a steel‐blue‐shaded cell. The rightmost column reports the total number of isolates in which each replicon was observed, and the bottom row summarizes the total replicon count identified per isolate.

## 4 Discussion

Phenotypic surveillance of bacterial infection in clinical settings complemented with genomic studies enables elucidation of the basis of disease cause, accurate identification, transmission dynamics, and AMR (Ahmed et al. 2024; Govender 2022). Our study highlights the need to adapt genetic surveillance to complement routine phenotypic investigations, especially for the accurate identification of bacteria and their resistance to antibiotics in resource‐limited settings. We noticed a discordance between phenotypic and genotypic identification of the bacterial isolates. For example, some *E. coli* and *K. Pneumoniae* were phenotypically misidentified as *Citrobacter spp* and an *E. cloaca* as *S. enterica*. Rosenthal et al. have posited that bacterial misidentification can arise from laboratory procedural errors, such as culture media contamination or the use of suboptimal reagents (e.g., dried agar and expired substrates), which skew biochemical test results (Rosenthal et al. 1978). Additionally, evolutionary mutations in bacteria, driven by selective pressures such as antibiotics or nutrient scarcity, can alter phenotypic changes via genetic mutations or horizontal gene transfer, thereby confounding precise taxonomic identification (Blount et al. 2020). Misidentification of bacteria in the clinical setting could result in deleterious clinical outcomes, a phenomenon that can be forestalled by complementary genomic profiling (Heyman et al. 2025; Liang 2020). Additionally, genomic characterization can reveal the genetic basis of AMRs even for drugs for which AST was not performed. Also, VFs and plasmid identities from genomic profiling can be used to predict the pathogenicity potential and transmissibility of AMRs, respectively (Heyman et al. 2025; Arber 2000).

ESBL‐producing *Enterobacteriaceae* have been implicated in the outbreak of several bacteria‐borne diseases, including food‐borne diseases and nosocomial and care‐home outbreaks (Ibrahim et al. 2023; Do Tran et al. 2022; van Bilsen et al. 2021). In our study, 93% of the previously suspected ESBL producers were confirmed to be ESBL producers. Some hospital‐based studies in Cape Coast, Ghana (Sampah et al. 2023), and other African countries (Asare Yeboah et al. 2024; Bezabih et al. 2022b; Fadare and Okoh 2021) have reported similar rates of ESBL‐producing *Enterobacteriaceae*, especially in *E. coli* and *K. pneumoniae*. However, a lower rate of 14.6% was observed in a hospital‐based study conducted in Ontario, Canada (Hasan et al. 2023). The ESBL‐producing isolates sequenced harbored 17 individual ß‐lactamase ARGs of Ambler groups A, B, C, and D. ß‐lactamases associated with ESBLs observed were mainly *SHV, TEM*, and *CTX‐M* type genes found in the Ambler group A. These variants are mostly penicillinases and cephalosporinases and tend to be resistant to clavulanate, sulbactam, and tazobactam (Sawa et al. 2020; Bush and Jacoby 2010). This could explain why the addition of clavulanate to ceftazidime did not improve the sensitivity profile during AST. *CTX‐M‐15*, a member of the Ambler class A, was the predominant ß‐lactamase ARG among the isolates, accounting for 68.4% (13/19). Previous studies in Ghana have reported *CTX‐M‐15* prevalence of over 80% in *Enterobacteriaceae* (Obeng‐Nkrumah et al. 2023; Eibach et al. 2016), whereas Asare Yeboah et al (Asare Yeboah et al. 2024) reported a lower proportion of 52%. Globally, CTX*‐M‐15* is the most prevalent ß‐lactamase ARG in *Enterobacteriaceae*, with a pooled prevalence of 16%, which is lower than 74% (14/19) observed in our study due to widespread dissemination of IncF plasmids that often carry this gene. We also observed the presence of oxacillinases, including OXA‐1, OXA‐58, and OXA‐402, which also confer ESBL activities (Sawa et al. 2020). Generally, most isolates were sensitive to carbapenems, with just above 11% of the isolates (9/79) phenotypically resistant to meropenem. However, only 2 of these resistant isolates, both *K. pneumoniae* (BC71 and BC75), were part of the whole genome sequenced isolates. These *K. pneumoniae* isolates harbored NDM‐1 carbapenemase. NDM‐1, an Ambler class B ß‐lactamase, has previously been described in *E. coli* clinical isolates and hospital environment surveillance studies (Ayibieke et al. 2018; Acolatse et al. 2022).

The majority of VFs identified in the WGS isolates were in adherence, effector delivery systems, and nutritional or metabolic factor families, mostly in *E. coli* and *K. pneumoniae*. However, only *S. enterica* harbored most effector delivery systems. Studies conducted in both Ghana and Slovakia (Bujňáková et al. 2022; Dekker et al. 2021) observed that the prevalence of these 3 VFs among *Enterobacteriaceae*, especially *E*. *coli*, *K. pneumoniae*, *E. cloacae*, and *S*. *enterica*, was high. Other studies in Africa and some European countries corroborate our findings (Leitão 2020; Dougnon et al. 2020; Hetsa et al. 2024; Amaretti et al. 2020). In our study, the whole genome sequenced isolates included 11 isolates, that is, 3 *K. pneumoniae* and 8 *E. coli*, which were isolated from urine infections. Generally, the ability of *Enterobacteriaceae* to colonize and cause urinary tract infections depends on their ability to form biofilms and produce several VFs, especially adherence factors (P fimbriae, S fimbriae, type I fimbriae), iron acquisition and sequestration (aerobactin and enterobactins), hemolysin, and cytotoxic necrotizing factors (cnf1) (Assouma et al. 2023; Govindarajan and Kandaswamy 2022).

Fimbriae are an absolute prerequisite for urinary tract colonization and disease causation, because *Enterobacteriaceae* uses that to adhere to host cells and form protective biofilms in the urinary tract, contributing to the pathogenesis and persistence of urinary tract infections (Connell et al. 1996; Hornick et al. 1991). Incidentally, the *E. coli* and *K. pneumoniae* sequenced possessed numerous fimbrial genes, including type 1, type 2 (P fimbriae), type 3, and S fimbriae. Specifically, *E. coli* harbored type I fimbriae (*fimA, B, I, D, E G, H, K, F, T, U, V*), P fimbriae (*papB, C, F, G, H, D, F, J, K, X)*, and S fimbriae (*sfaD, E, F, C, G, H, S, X, Y)*. Studies in uropathogenic *Enterobacteriaceae* conducted in Benin, Romania, Mongolia, and Egypt have also reported these fimbrial genes (Assouma et al. 2023). Uropathogenic *K. pneumoniae* uses two main types of fimbriae for adhesion to the uroepithelium, type 1 and type III (Stahlhut et al. 2012). However, we observed type II fimbriae (*papF, papK*) in the 3 uropathogenic *K. pneumoniae* isolates in addition to type I fimbriae (*fimA, B, D, E, F, G, H, I, K*) and type III fimbriae (*mrkC, I, F, A, D, B, H, D*).

In addition to these adherence factors, *E. coli* isolates also harbored iron acquisition and sequestration genes, including aerobactins (*iucA, iucB, iutA*) and enterobactins (*entA, entC, entE, entF, entS, entB*). The 3 *K. pneumoniae* also possessed an aerobactin (*iutA*.) and enterobactins (*entF, entA, entE, entC, entS*). Siderophores, such as enterobactins and aerobactins, enable uropathogenic *Enterobacteriaceae* to thrive in the host by scavenging iron from the host epithelial cells (Qi and Han 2018). According to Moxley et al. (Payne and Neilands 1988; Moxley 2022), *Enterobacteriaceae* requires iron for growth and virulence. Hemolysin (*hlyC, hlyD*) and *cnf1*, which *E. coli* uses to damage cell nutrients and trigger siderophores to sequester iron from the host for growth (Sung et al. 2024), were also observed among the *E. coli* isolates. Nhu et al. reported that hemolysins (*hlyC, D*) and cnf1 were found abundantly in UPEC strains, emphasizing their significance capacity to cause UTI (Nhu et al. 2019).

According to Nordmann and Poirel (2005); Algarni et al. (2024), most ARGs and VFs are borne on mobile genetic elements, especially plasmids, and have become important for the dissemination of these factors within the *Enterobacteriaceae* family through horizontal or vertical gene transfer (Rozwandowicz et al. 2018; Atala 2020; Ma et al. 2023; Algarni et al. 2024). It has been reported that most plasmid replicons found in *Enterobacteriaceae* are I‐complex (Incompatibility complex) plasmids and are mostly resistance and fertility plasmids in addition to colicinogenic plasmid (Carattoli et al. 2021; Rozwandowicz et al. 2018; Yu et al. 2022 Nov 16; Benz et al. 2021). In our study, the I‐complex we observed most was the IncF (13/19 total Inc plasmids) (Rozwandowicz et al. 2018; Naseer and Sundsfjord 2011; Stein et al. 2024). These plasmids have been reported worldwide for the dissemination of AMR genes, especially ESBLs, and particularly *CTX‐M‐15* (Rozwandowicz et al. 2018; Naseer and Sundsfjord 2011; Stein et al. 2024). Unsurprisingly, *CTX‐M‐15* was the most common ARG identified in our study. In our study, these complex groups of plasmid replicons were observed in *E. coli and K. pneumoniae*, similar to previous reports (Rozwandowicz et al. 2018; Stein et al. 2024). Other Inc plasmid replicons observed in our study included *IncC, IncH, IncI, IncR*, and *IncX*.

Consistent with previous investigations, our study also adds to the expanding body of evidence indicating that colicins co‐harbor with ARGs to facilitate AMR co‐selection and dissemination (Marković et al. 2022b; Daniels A. O et al. 2023; Chérier et al. 2021).

This study characterizes the resistome and virulome of *Enterobacteriaceae* isolates at CCTH, highlighting the pressing threat posed by ESBL‐producing *Enterobacteriaceae* to healthcare delivery. Furthermore, it highlights plasmid replicons associated with AMR gene transmission, emphasizing the role of genomic data in informing accurate diagnosis and rational antibiotic use.

## 5 Conclusions

Our study revealed alarming levels of MDR determinants, particularly against β‐lactam antibiotics, in the isolates. This represents one of the first in‐depth genomic assessments of ESBL‐producing *Enterobacteriaceae* in Ghana. Nearly all isolates were confirmed to be ESBL producers. Genomic analyses confirmed that the selected isolates were ESBL gene carriers. It also revealed 139 distinct resistance determinants across diverse classes of drugs, especially β‐lactams. CTX‐M‐15 β‐lactamase was the predominant ESBL gene, and NDM‐1 carbapenemase was detected in *K. pneumoniae* isolates, signaling the emergence of carbapenem resistance at CCTH. These pathogens harbored numerous VFs (fimbrial adhesins, siderophores, hemolysins, and secretion system components) and diverse plasmid replicons (especially, IncF and colicin plasmids), facilitating resistance dissemination. In aggregate, these findings provide robust genomic evidence of pervasive ESBL‐mediated resistance and emergent carbapenemase activity in Ghanaian *Enterobacteriaceae*, highlighting the urgent need for enhanced antimicrobial stewardship, routine genomic surveillance, and robust infection control measures to mitigate this growing public health threat in the Cape Coast Metropolis and other parts of Ghana.

## 6 Limitations

The phenotypic resistance assessment was limited to disc diffusion methods, mainly constrained by logistical limitations. Our findings potentially do not fully capture the genetic assessment of the plasmids of the individual isolates due to the usage of Illumina short reads, which could lead to incomplete reconstructions of plasmids or misassembles. It should be noted that our study circumscribed only those *Enterobacteriaceae* isolates accessible from the bacteriology laboratory at CCTH and with the availability of socio‐demographic and clinical data. Thus, our phenotypic AMR assessment does not fully capture the available *Enterobacteriaceae* isolates present at the time of this study at the hospital. Also due to funding constraints only 20 out of the 100 isolates were whole genome sequenced; our findings potentially do not fully capture the AMR genotypic assessment of all the retrieved isolates.

## Author Contributions

**Bismark Donkor:** conceptualization; methodology; data curation; investigation; formal analysis; visualization; writing – review and editing; writing – original draft; software; validation. **Richael Odarkor Mills:** methodology; investigation; writing – review and editing; validation; formal analysis. **Philimon Mwintige:** investigation; methodology. **Alberta Bedford Moses:** investigation; methodology. **Abigail Asmah Brown:** investigation; methodology. **Faustina Halm‐Lai:** conceptualization; writing – review and editing; supervision; investigation; project administration; resources. **Oheneba Charles Kofi Hagan:** conceptualization; investigation; funding acquisition; writing – original draft; writing – review and editing; visualization; validation; methodology; formal analysis; project administration; supervision; data curation; resources; software.

## Ethics Statement

Ethical approval for this study was obtained from the Cape Coast Teaching Hospital Ethical Clearance Review Committee (CCTHERC), Cape Coast, Ghana, under the approval number CCTHERC/EC/2023/102. To ensure privacy and confidentiality, data were retrieved deidentified.

## Conflicts of Interest

The authors declare that there are no personal relationships that could have influenced the work reported in this study.

## Supporting information

Table A1.

Table A2.

Table A3.

Table A4.

Table A5.

## Data Availability

The raw sequencing data generated in this study have been deposited in the NCBI Sequence Read 408 Archive (SRA) under Bio Project accession number PRJNA1271415.
