# Supplementary material for: Phenotypic and Genotypic Profile of Enterobacteriaceae Isolated at a Teaching Hospital in Ghana
Source: Microbiologyopen. 2025 Nov 9;14(6):e70127. doi: 10.1002/mbo3.70127 (PMC12597788; doi:10.1002/mbo3.70127)
Supplement: Supplementary file 3 — Table A3. [file MBO3-14-e70127-s004.docx]

**Table A3**: Presence of antimicrobial resistance genes (ARGs) in *Enterobacteriaceae* bacterial isolates

| **ARGs** | | ***E. coli*** | | | | | | | | ***K. Pneumoniae*** | | | | | | | | ***E. cloacae*** | | ***S. enterica*** | **Number of Isolates** |
| --- | --- | --- | --- | --- | --- | --- | --- | --- | --- | --- | --- | --- | --- | --- | --- | --- | --- | --- | --- | --- | --- |
|  | | **BC04** | **BC15** | **BC18** | **BC26** | **BC64** | **BC73** | **BC81** | **BC82** | **BC16** | **BC20** | **BC37** | **BC54** | **BC71** | **BC75** | **BC76** | **BC94** | **BC06** | **BC35** | **BC38** |  |
| **Aminoglycosides** | | | | | | | | | | | | | | | | | | | | | |
| *AAC (3')-IId* | |  |  |  |  |  |  |  |  |  |  |  |  |  |  |  |  |  |  |  | **4** |
| *AAC (3')-IIe* | |  |  |  |  |  |  |  |  |  |  |  |  |  |  |  |  |  |  |  | **5** |
| *AAC (6')-Iy* | |  |  |  |  |  |  |  |  |  |  |  |  |  |  |  |  |  |  |  | **1** |
| *AAD (3'')-Ib* | |  |  |  |  |  |  |  |  |  |  |  |  |  |  |  |  |  |  |  | **6** |
| *AAD (3')-VIb* | |  |  |  |  |  |  |  |  |  |  |  |  |  |  |  |  |  |  |  | **5** |
| *AAD (6)-Id* | |  |  |  |  |  |  |  |  |  |  |  |  |  |  |  |  |  |  |  | **5** |
| *AAC (3')-Via* | |  |  |  |  |  |  |  |  |  |  |  |  |  |  |  |  |  |  |  | **5** |
| *aacA2* | |  |  |  |  |  |  |  |  |  |  |  |  |  |  |  |  |  |  |  | **2** |
| *aadA5* | |  |  |  |  |  |  |  |  |  |  |  |  |  |  |  |  |  |  |  | **7** |
| *aadA2* | |  |  |  |  |  |  |  |  |  |  |  |  |  |  |  |  |  |  |  | **4** |
| *armA* | |  |  |  |  |  |  |  |  |  |  |  |  |  |  |  |  |  |  |  | **3** |
| *AAC (6')-Ib-cr6* | |  |  |  |  |  |  |  |  |  |  |  |  |  |  |  |  |  |  |  | **11** |
| *acrB* | |  |  |  |  |  |  |  |  |  |  |  |  |  |  |  |  |  |  |  | **9** |
| *acrD* | |  |  |  |  |  |  |  |  |  |  |  |  |  |  |  |  |  |  |  | **9** |
| *AcrE* | |  |  |  |  |  |  |  |  |  |  |  |  |  |  |  |  |  |  |  | **9** |
| *AcrF* | |  |  |  |  |  |  |  |  |  |  |  |  |  |  |  |  |  |  |  | **9** |
| *arr-3* | |  |  |  |  |  |  |  |  |  |  |  |  |  |  |  |  |  |  |  | **2** |
| *ArnT* | |  |  |  |  |  |  |  |  |  |  |  |  |  |  |  |  |  |  |  | **6** |
| *cpxA* | |  |  |  |  |  |  |  |  |  |  |  |  |  |  |  |  |  |  |  | **9** |
| *Klebsiella_pneumoniae_KpnE* | |  |  |  |  |  |  |  |  |  |  |  |  |  |  |  |  |  |  |  | **7** |
| *Klebsiella_pneumoniae_KpnF* | |  |  |  |  |  |  |  |  |  |  |  |  |  |  |  |  |  |  |  | **8** |
| *Klebsiella_pneumoniae_KpnG* | |  |  |  |  |  |  |  |  |  |  |  |  |  |  |  |  |  |  |  | **7** |
| *kdpE* | |  |  |  |  |  |  |  |  |  |  |  |  |  |  |  |  |  |  |  | **8** |
| **Fluoroquinolones** | | | | | | | | | | | | | | | | | | | | | |
| *AAC (6')-Ib-cr6* | |  |  |  |  |  |  |  |  |  |  |  |  |  |  |  |  |  |  |  | **11** |
| *abeM* | |  |  |  |  |  |  |  |  |  |  |  |  |  |  |  |  |  |  |  | **2** |
| *abeS* | |  |  |  |  |  |  |  |  |  |  |  |  |  |  |  |  |  |  |  | **2** |
| *oqxA* |  |  |  |  |  |  |  |  |  |  |  |  |  |  |  |  |  |  |  |  | **6** |
| *oqxB* |  |  |  |  |  |  |  |  |  |  |  |  |  |  |  |  |  |  |  |  | **7** |
| *QnrB17* | |  |  |  |  |  |  |  |  |  |  |  |  |  |  |  |  |  |  |  | **5** |
| *QnrD1* | |  |  |  |  |  |  |  |  |  |  |  |  |  |  |  |  |  |  |  | **1** |
| *QnrS1* | |  |  |  |  |  |  |  |  |  |  |  |  |  |  |  |  |  |  |  | **2** |
| *adeF* | |  |  |  |  |  |  |  |  |  |  |  |  |  |  |  |  |  |  |  | **2** |
| *adeG* | |  |  |  |  |  |  |  |  |  |  |  |  |  |  |  |  |  |  |  | **2** |
| *adeH* | |  |  |  |  |  |  |  |  |  |  |  |  |  |  |  |  |  |  |  | **2** |
| *adeI* | |  |  |  |  |  |  |  |  |  |  |  |  |  |  |  |  |  |  |  | **2** |
| *adeJ* | |  |  |  |  |  |  |  |  |  |  |  |  |  |  |  |  |  |  |  | **2** |
| *adeK* | |  |  |  |  |  |  |  |  |  |  |  |  |  |  |  |  |  |  |  | **2** |
| *adeL* | |  |  |  |  |  |  |  |  |  |  |  |  |  |  |  |  |  |  |  | **2** |
| *adeN* | |  |  |  |  |  |  |  |  |  |  |  |  |  |  |  |  |  |  |  | **2** |
| *Acinetobacter_baumannii_AbaF* | |  |  |  |  |  |  |  |  |  |  |  |  |  |  |  |  |  |  |  | **2** |
| *Acinetobacter_baumannii_AmvA* | |  |  |  |  |  |  |  |  |  |  |  |  |  |  |  |  |  |  |  | **2** |
| *acrB* | |  |  |  |  |  |  |  |  |  |  |  |  |  |  |  |  |  |  |  | **9** |
| *acrD* | |  |  |  |  |  |  |  |  |  |  |  |  |  |  |  |  |  |  |  | **9** |
| *AcrE* | |  |  |  |  |  |  |  |  |  |  |  |  |  |  |  |  |  |  |  | **8** |
| *AcrF* | |  |  |  |  |  |  |  |  |  |  |  |  |  |  |  |  |  |  |  | **9** |
| *AcrS* | |  |  |  |  |  |  |  |  |  |  |  |  |  |  |  |  |  |  |  | **9** |
| *bacA* | |  |  |  |  |  |  |  |  |  |  |  |  |  |  |  |  |  |  |  | **10** |
| *CRP* | |  |  |  |  |  |  |  |  |  |  |  |  |  |  |  |  |  |  |  | **9** |
| *eptA* | |  |  |  |  |  |  |  |  |  |  |  |  |  |  |  |  |  |  |  | **4** |
| *ermA* | |  |  |  |  |  |  |  |  |  |  |  |  |  |  |  |  |  |  |  | **8** |
| *ermB* | |  |  |  |  |  |  |  |  |  |  |  |  |  |  |  |  |  |  |  | **9** |
| *ermK* | |  |  |  |  |  |  |  |  |  |  |  |  |  |  |  |  |  |  |  | **7** |
| *ermR* | |  |  |  |  |  |  |  |  |  |  |  |  |  |  |  |  |  |  |  | **8** |
| *ermY* | |  |  |  |  |  |  |  |  |  |  |  |  |  |  |  |  |  |  |  | **7** |
| *Escherichia_coli_emrE* | |  |  |  |  |  |  |  |  |  |  |  |  |  |  |  |  |  |  |  | **1** |
| *Escherichia_coli_mdfA* | |  |  |  |  |  |  |  |  |  |  |  |  |  |  |  |  |  |  |  | **9** |
| *evgA* | |  |  |  |  |  |  |  |  |  |  |  |  |  |  |  |  |  |  |  | **7** |
| *evgS* | |  |  |  |  |  |  |  |  |  |  |  |  |  |  |  |  |  |  |  | **7** |
| *gadX* | |  |  |  |  |  |  |  |  |  |  |  |  |  |  |  |  |  |  |  | **5** |
| *marA* | |  |  |  |  |  |  |  |  |  |  |  |  |  |  |  |  |  |  |  | **9** |
| *mdtA* | |  |  |  |  |  |  |  |  |  |  |  |  |  |  |  |  |  |  |  | **9** |
| *mdtB* | |  |  |  |  |  |  |  |  |  |  |  |  |  |  |  |  |  |  |  | **9** |
| *mdtC* | |  |  |  |  |  |  |  |  |  |  |  |  |  |  |  |  |  |  |  | **8** |
| *mdtE* | |  |  |  |  |  |  |  |  |  |  |  |  |  |  |  |  |  |  |  | **9** |
| *mdtF* | |  |  |  |  |  |  |  |  |  |  |  |  |  |  |  |  |  |  |  | **9** |
| *mdtH* | |  |  |  |  |  |  |  |  |  |  |  |  |  |  |  |  |  |  |  | **9** |
| *MdtK* | |  |  |  |  |  |  |  |  |  |  |  |  |  |  |  |  |  |  |  | **1** |
| *sdiA* | |  |  |  |  |  |  |  |  |  |  |  |  |  |  |  |  |  |  |  | **1** |
| **ß-Lactams** | | | | | | | | | | | | | | | | | | | | | |
| *ADC-169* | |  |  |  |  |  |  |  |  |  |  |  |  |  |  |  |  |  |  |  | **2** |
| *CMH-3* | |  |  |  |  |  |  |  |  |  |  |  |  |  |  |  |  |  |  |  | **1** |
| *CMH-6* | |  |  |  |  |  |  |  |  |  |  |  |  |  |  |  |  |  |  |  | **2** |
| *CMY-59* | |  |  |  |  |  |  |  |  |  |  |  |  |  |  |  |  |  |  |  | **1** |
| *CTX-M-15* | |  |  |  |  |  |  |  |  |  |  |  |  |  |  |  |  |  |  |  | **13** |
| *EC-5* | |  |  |  |  |  |  |  |  |  |  |  |  |  |  |  |  |  |  |  | **4** |
| *EC-15* | |  |  |  |  |  |  |  |  |  |  |  |  |  |  |  |  |  |  |  | **1** |
| *Escherichia_coli_ampC_beta-lactamase* | |  |  |  |  |  |  |  |  |  |  |  |  |  |  |  |  |  |  |  | **3** |
| *NDM-1* | |  |  |  |  |  |  |  |  |  |  |  |  |  |  |  |  |  |  |  | **4** |
| *OXA-1* | |  |  |  |  |  |  |  |  |  |  |  |  |  |  |  |  |  |  |  | **8** |
| *OXA-402* | |  |  |  |  |  |  |  |  |  |  |  |  |  |  |  |  |  |  |  | **2** |
| *OXA-58* | |  |  |  |  |  |  |  |  |  |  |  |  |  |  |  |  |  |  |  | **2** |
| *SHV-106* | |  |  |  |  |  |  |  |  |  |  |  |  |  |  |  |  |  |  |  | **4** |
| *SHV-80* | |  |  |  |  |  |  |  |  |  |  |  |  |  |  |  |  |  |  |  | **1** |
| *SHV-187* | |  |  |  |  |  |  |  |  |  |  |  |  |  |  |  |  |  |  |  | **3** |
| *TEM-1* | |  |  |  |  |  |  |  |  |  |  |  |  |  |  |  |  |  |  |  | **6** |
| *TEM-35* | |  |  |  |  |  |  |  |  |  |  |  |  |  |  |  |  |  |  |  | **1** |
| *adeI* | |  |  |  |  |  |  |  |  |  |  |  |  |  |  |  |  |  |  |  | **2** |
| *adeJ* | |  |  |  |  |  |  |  |  |  |  |  |  |  |  |  |  |  |  |  | **2** |
| *adeK* | |  |  |  |  |  |  |  |  |  |  |  |  |  |  |  |  |  |  |  | **2** |
| *adeL* | |  |  |  |  |  |  |  |  |  |  |  |  |  |  |  |  |  |  |  | **2** |
| *adeN* | |  |  |  |  |  |  |  |  |  |  |  |  |  |  |  |  |  |  |  | **2** |
| *Acinetobacter_baumannii_AbaF* | |  |  |  |  |  |  |  |  |  |  |  |  |  |  |  |  |  |  |  | **2** |
| *Acinetobacter_baumannii_AmvA* | |  |  |  |  |  |  |  |  |  |  |  |  |  |  |  |  |  |  |  | **2** |
| *acrB* | |  |  |  |  |  |  |  |  |  |  |  |  |  |  |  |  |  |  |  | **9** |
| *acrD* | |  |  |  |  |  |  |  |  |  |  |  |  |  |  |  |  |  |  |  | **9** |
| *AcrE* | |  |  |  |  |  |  |  |  |  |  |  |  |  |  |  |  |  |  |  | **9** |
| *AcrF* | |  |  |  |  |  |  |  |  |  |  |  |  |  |  |  |  |  |  |  | **9** |
| *AcrS* | |  |  |  |  |  |  |  |  |  |  |  |  |  |  |  |  |  |  |  | **9** |
| *bacA* | |  |  |  |  |  |  |  |  |  |  |  |  |  |  |  |  |  |  |  | **10** |
| *CRP* | |  |  |  |  |  |  |  |  |  |  |  |  |  |  |  |  |  |  |  | **9** |
| *eptA* | |  |  |  |  |  |  |  |  |  |  |  |  |  |  |  |  |  |  |  | **4** |
| *Erm (49)* | |  |  |  |  |  |  |  |  |  |  |  |  |  |  |  |  |  |  |  | **1** |
| *Escherichia_coli_acrA* | |  |  |  |  |  |  |  |  |  |  |  |  |  |  |  |  |  |  |  | **9** |
| *Escherichia_coli_emrE* | |  |  |  |  |  |  |  |  |  |  |  |  |  |  |  |  |  |  |  | **1** |
| *Escherichia_coli_mdfA* | |  |  |  |  |  |  |  |  |  |  |  |  |  |  |  |  |  |  |  | **9** |
| *evgA* | |  |  |  |  |  |  |  |  |  |  |  |  |  |  |  |  |  |  |  | **7** |
| *evgS* | |  |  |  |  |  |  |  |  |  |  |  |  |  |  |  |  |  |  |  | **7** |
| *gadW* | |  |  |  |  |  |  |  |  |  |  |  |  |  |  |  |  |  |  |  | **3** |
| *gadX* | |  |  |  |  |  |  |  |  |  |  |  |  |  |  |  |  |  |  |  | **5** |
| *golS* | |  |  |  |  |  |  |  |  |  |  |  |  |  |  |  |  |  |  |  | **1** |
| *H-NS* | |  |  |  |  |  |  |  |  |  |  |  |  |  |  |  |  |  |  |  | **8** |
| *Klebsiella_pneumoniae_KpnE* | |  |  |  |  |  |  |  |  |  |  |  |  |  |  |  |  |  |  |  | **7** |
| *Klebsiella_pneumoniae_KpnF* | |  |  |  |  |  |  |  |  |  |  |  |  |  |  |  |  |  |  |  | **8** |
| *Klebsiella_pneumoniae_KpnG* | |  |  |  |  |  |  |  |  |  |  |  |  |  |  |  |  |  |  |  | **7** |
| *LptD* | |  |  |  |  |  |  |  |  |  |  |  |  |  |  |  |  |  |  |  | **6** |
| *LpsB* | |  |  |  |  |  |  |  |  |  |  |  |  |  |  |  |  |  |  |  | **2** |
| *marA* | |  |  |  |  |  |  |  |  |  |  |  |  |  |  |  |  |  |  |  | **9** |
| *mdsA* | |  |  |  |  |  |  |  |  |  |  |  |  |  |  |  |  |  |  |  | **10** |
| *mdsB* | |  |  |  |  |  |  |  |  |  |  |  |  |  |  |  |  |  |  |  | **10** |
| *mdsC* | |  |  |  |  |  |  |  |  |  |  |  |  |  |  |  |  |  |  |  | **1** |
| *mdtB* | |  |  |  |  |  |  |  |  |  |  |  |  |  |  |  |  |  |  |  | **9** |
| *mdtC* | |  |  |  |  |  |  |  |  |  |  |  |  |  |  |  |  |  |  |  | **8** |
| *mdtE* | |  |  |  |  |  |  |  |  |  |  |  |  |  |  |  |  |  |  |  | **9** |
| *mdtF* | |  |  |  |  |  |  |  |  |  |  |  |  |  |  |  |  |  |  |  | **9** |
| *mdtN* | |  |  |  |  |  |  |  |  |  |  |  |  |  |  |  |  |  |  |  | **9** |
| *mdtO* | |  |  |  |  |  |  |  |  |  |  |  |  |  |  |  |  |  |  |  | **9** |
| *mdtP* | |  |  |  |  |  |  |  |  |  |  |  |  |  |  |  |  |  |  |  | **9** |
| *MdtQ* | |  |  |  |  |  |  |  |  |  |  |  |  |  |  |  |  |  |  |  | **8** |
| *OmpA* | |  |  |  |  |  |  |  |  |  |  |  |  |  |  |  |  |  |  |  | **8** |
| *ramA* | |  |  |  |  |  |  |  |  |  |  |  |  |  |  |  |  |  |  |  | **3** |
| *SAT-2* | |  |  |  |  |  |  |  |  |  |  |  |  |  |  |  |  |  |  |  | **1** |
| *sdiA* | |  |  |  |  |  |  |  |  |  |  |  |  |  |  |  |  |  |  |  | **1** |
| *TolC* | |  |  |  |  |  |  |  |  |  |  |  |  |  |  |  |  |  |  |  | **9** |
| **Sulphonamides** | | | | | | | | | | | | | | | | | | | | | |
| *sul1* | |  |  |  |  |  |  |  |  |  |  |  |  |  |  |  |  |  |  |  | **10** |
| *sul2* | |  |  |  |  |  |  |  |  |  |  |  |  |  |  |  |  |  |  |  | **10** |
| *CRP* | |  |  |  |  |  |  |  |  |  |  |  |  |  |  |  |  |  |  |  | **9** |
| *dfrA1* | |  |  |  |  |  |  |  |  |  |  |  |  |  |  |  |  |  |  |  | **1** |
| *dfrA12* | |  |  |  |  |  |  |  |  |  |  |  |  |  |  |  |  |  |  |  | **4** |
| *dfrA14* | |  |  |  |  |  |  |  |  |  |  |  |  |  |  |  |  |  |  |  | **4** |
| *dfrA17* | |  |  |  |  |  |  |  |  |  |  |  |  |  |  |  |  |  |  |  | **6** |
| *dfrA27* | |  |  |  |  |  |  |  |  |  |  |  |  |  |  |  |  |  |  |  | **1** |
| *eptA* | |  |  |  |  |  |  |  |  |  |  |  |  |  |  |  |  |  |  |  | **4** |
| **Chloramphenicol** | | | | | | | | | | | | | | | | | | | | | |
| *catA1* | |  |  |  |  |  |  |  |  |  |  |  |  |  |  |  |  |  |  |  | **5** |
| *catA4* | |  |  |  |  |  |  |  |  |  |  |  |  |  |  |  |  |  |  |  | **1** |
| *catII_from_Escherichia_coli_K-12* | |  |  |  |  |  |  |  |  |  |  |  |  |  |  |  |  |  |  |  | **1** |
| *floR* | |  |  |  |  |  |  |  |  |  |  |  |  |  |  |  |  |  |  |  | **2** |
| *adeH* | |  |  |  |  |  |  |  |  |  |  |  |  |  |  |  |  |  |  |  | **2** |
| *adeI* | |  |  |  |  |  |  |  |  |  |  |  |  |  |  |  |  |  |  |  | **2** |
| *adeJ* | |  |  |  |  |  |  |  |  |  |  |  |  |  |  |  |  |  |  |  | **2** |
| *adeK* | |  |  |  |  |  |  |  |  |  |  |  |  |  |  |  |  |  |  |  | **2** |
| *adeN* | |  |  |  |  |  |  |  |  |  |  |  |  |  |  |  |  |  |  |  | **2** |
| *Acinetobacter_baumannii_AbaF* | |  |  |  |  |  |  |  |  |  |  |  |  |  |  |  |  |  |  |  | **2** |
| *Acinetobacter_baumannii_AmvA* | |  |  |  |  |  |  |  |  |  |  |  |  |  |  |  |  |  |  |  | **2** |
| *acrB* | |  |  |  |  |  |  |  |  |  |  |  |  |  |  |  |  |  |  |  | **9** |
| *acrD* | |  |  |  |  |  |  |  |  |  |  |  |  |  |  |  |  |  |  |  | **9** |
| *AcrE* | |  |  |  |  |  |  |  |  |  |  |  |  |  |  |  |  |  |  |  | **9** |
| *AcrF* | |  |  |  |  |  |  |  |  |  |  |  |  |  |  |  |  |  |  |  | **9** |
| *FosA6* | |  |  |  |  |  |  |  |  |  |  |  |  |  |  |  |  |  |  |  | **7** |
| *golS* | |  |  |  |  |  |  |  |  |  |  |  |  |  |  |  |  |  |  |  | **1** |
| *marA* | |  |  |  |  |  |  |  |  |  |  |  |  |  |  |  |  |  |  |  | **9** |
| *mdsA* | |  |  |  |  |  |  |  |  |  |  |  |  |  |  |  |  |  |  |  | **10** |
| *mdsB* | |  |  |  |  |  |  |  |  |  |  |  |  |  |  |  |  |  |  |  | **10** |
| *mdsC* | |  |  |  |  |  |  |  |  |  |  |  |  |  |  |  |  |  |  |  | **1** |
| *mdtG* | |  |  |  |  |  |  |  |  |  |  |  |  |  |  |  |  |  |  |  | **9** |
| *mdtM* | |  |  |  |  |  |  |  |  |  |  |  |  |  |  |  |  |  |  |  | **6** |
| *mdtN* | |  |  |  |  |  |  |  |  |  |  |  |  |  |  |  |  |  |  |  | **9** |
| *mdtO* | |  |  |  |  |  |  |  |  |  |  |  |  |  |  |  |  |  |  |  | **9** |
| *mdtP* | |  |  |  |  |  |  |  |  |  |  |  |  |  |  |  |  |  |  |  | **9** |
| *MdtQ* | |  |  |  |  |  |  |  |  |  |  |  |  |  |  |  |  |  |  |  | **7** |
| *sdiA* | |  |  |  |  |  |  |  |  |  |  |  |  |  |  |  |  |  |  |  | **1** |
| **Tetracyclines** | | | | | | | | | | | | | | | | | | | | | |
| *tet(A)* | |  |  |  |  |  |  |  |  |  |  |  |  |  |  |  |  |  |  |  | **8** |
| *tet(B)* | |  |  |  |  |  |  |  |  |  |  |  |  |  |  |  |  |  |  |  | **4** |
| *tet(D)* | |  |  |  |  |  |  |  |  |  |  |  |  |  |  |  |  |  |  |  | **2** |
| *tet (39)* | |  |  |  |  |  |  |  |  |  |  |  |  |  |  |  |  |  |  |  | **2** |
| *adeA* | |  |  |  |  |  |  |  |  |  |  |  |  |  |  |  |  |  |  |  | **2** |
| *adeB* | |  |  |  |  |  |  |  |  |  |  |  |  |  |  |  |  |  |  |  | **2** |
| *adeF* | |  |  |  |  |  |  |  |  |  |  |  |  |  |  |  |  |  |  |  | **2** |
| *adeG* | |  |  |  |  |  |  |  |  |  |  |  |  |  |  |  |  |  |  |  | **2** |
| *adeH* | |  |  |  |  |  |  |  |  |  |  |  |  |  |  |  |  |  |  |  | **2** |
| *adeI* | |  |  |  |  |  |  |  |  |  |  |  |  |  |  |  |  |  |  |  | **2** |
| *adeJ* | |  |  |  |  |  |  |  |  |  |  |  |  |  |  |  |  |  |  |  | **2** |
| *adeK* | |  |  |  |  |  |  |  |  |  |  |  |  |  |  |  |  |  |  |  | **2** |
| *adeL* | |  |  |  |  |  |  |  |  |  |  |  |  |  |  |  |  |  |  |  | **2** |
| *adeN* | |  |  |  |  |  |  |  |  |  |  |  |  |  |  |  |  |  |  |  | **2** |
| *adeR* | |  |  |  |  |  |  |  |  |  |  |  |  |  |  |  |  |  |  |  | **2** |
| *adeS* | |  |  |  |  |  |  |  |  |  |  |  |  |  |  |  |  |  |  |  | **2** |
| *acrB* | |  |  |  |  |  |  |  |  |  |  |  |  |  |  |  |  |  |  |  | **9** |
| *acrD* | |  |  |  |  |  |  |  |  |  |  |  |  |  |  |  |  |  |  |  | **9** |
| *AcrE* | |  |  |  |  |  |  |  |  |  |  |  |  |  |  |  |  |  |  |  | **8** |
| *AcrF* | |  |  |  |  |  |  |  |  |  |  |  |  |  |  |  |  |  |  |  | **9** |
| *AcrS* | |  |  |  |  |  |  |  |  |  |  |  |  |  |  |  |  |  |  |  | **9** |
| *ermA* | |  |  |  |  |  |  |  |  |  |  |  |  |  |  |  |  |  |  |  | **8** |
| *ermB* | |  |  |  |  |  |  |  |  |  |  |  |  |  |  |  |  |  |  |  | **9** |
| *ermK* | |  |  |  |  |  |  |  |  |  |  |  |  |  |  |  |  |  |  |  | **7** |
| *ermR* | |  |  |  |  |  |  |  |  |  |  |  |  |  |  |  |  |  |  |  | **8** |
| *ermY* | |  |  |  |  |  |  |  |  |  |  |  |  |  |  |  |  |  |  |  | **7** |
| *evgA* | |  |  |  |  |  |  |  |  |  |  |  |  |  |  |  |  |  |  |  | **7** |
| *evgS* | |  |  |  |  |  |  |  |  |  |  |  |  |  |  |  |  |  |  |  | **7** |
| *H-NS* | |  |  |  |  |  |  |  |  |  |  |  |  |  |  |  |  |  |  |  | **8** |
| *Klebsiella_pneumoniae_acrA* | |  |  |  |  |  |  |  |  |  |  |  |  |  |  |  |  |  |  |  | **7** |
| *Klebsiella_pneumoniae_KpnE* | |  |  |  |  |  |  |  |  |  |  |  |  |  |  |  |  |  |  |  | **7** |
| *Klebsiella_pneumoniae_KpnF* | |  |  |  |  |  |  |  |  |  |  |  |  |  |  |  |  |  |  |  | **8** |
| *Klebsiella_pneumoniae_KpnG* | |  |  |  |  |  |  |  |  |  |  |  |  |  |  |  |  |  |  |  | **7** |
| *marA* | |  |  |  |  |  |  |  |  |  |  |  |  |  |  |  |  |  |  |  | **9** |
| *sdiA* | |  |  |  |  |  |  |  |  |  |  |  |  |  |  |  |  |  |  |  | **1** |

The table presents the presence of various antimicrobial resistance genes (ARGs) across different isolates of *E. coli, K. pneumoniae, E. cloacae,* and *S. enterica*. Cells highlighted steel blue indicates the presence of gene in an isolate while cells with white colours indicate absence of genes. The numbers in the far-right column indicate the total number of isolates in which each gene was detected. They are categorized base on the class of drugs they conferred resistance. The colour code in the ARGs columns shows which class the genes present in the isolates conferred resistance on: Aminoglycosides [green], fluroquinolones [light green], ß-lactams [dark blue], sulphonamides [yellow], chloramphenicol [dark red], tetracycline [purple], and non-specific ARGs (multiple drug/class) [gold].

.
